# Nodular melanoma presenting with rapid progression and widespread metastases: a case report

**DOI:** 10.1186/1752-1947-3-50

**Published:** 2009-02-06

**Authors:** Mehmet Ali Erkurt, Ismet Aydogdu, Irfan Kuku, Emin Kaya, Yalcin Basaran

**Affiliations:** 1 Inonu University Faculty of Medicine, Department of Hematology, Turgut Ozal Medical Center, TR-44069 Malatya, Turkey; 2 Gulhane Military Medical Academy, Department of Internal Medicine, Ankara, Turkey

## Abstract

**Introduction:**

Melanoma is responsible for 1% to 2% of all cancer deaths around the world. Nodular melanoma often carries a poor prognosis because of no prodromal radial growth phase, early distant metastasis and significant tumor volume.

**Case presentation:**

We present a case of progressive melanoma. A 51-year-old man was admitted to our hospital with dyspnea and skin lesions. These were multiple, dark colored, firm, and nodular and varied in size. He was diagnosed with melanoma. Temozolomide was administered, but he died of respiratory failure within a week after diagnosis.

**Conclusion:**

Nodular melanoma tends to spread rapidly and eventually metastasize to vital organs. It may be fatal within months of recognition.

## Introduction

Melanoma is a neoplasm derived from melanocytes of the skin and other sites. It accounts for 1% to 3% of all malignancies and 1% to 2% of all cancer deaths worldwide. Recently, melanoma has become a major health problem in many countries. The worldwide incidence rate is increasing much more rapidly than for any other malignancies [1]. The mortality and morbidity rate from melanoma has risen about 2% annually since 1960 [2]. Projections were for 59,940 cases of melanoma and 48,290 cases of in situ melanoma to be newly diagnosed in the USA in 2007. Of these, 8110 cases were expected to be fatal [3].

The lifetime risk of melanoma is 1:70 in the population of the USA and will probably be 1:50 in 2010 [1]. According to the American Joint Committee on Cancer, melanomas are classified as superficial spreading melanoma, nodular melanoma, lentigo melanoma, acral lentiginous melanoma and unclassified melanoma. Nodular melanoma, comprising 10% to 15% of cutaneous melanomas, is the second most common variety of melanocytic neoplasms and occurs less commonly than superficial spreading melanoma. The median age of onset is 49 years. The duration of lesions before diagnosis is relatively short, ranging from a few months to 2 years [4]. Nodular melanoma often presents as an expanding darkly pigmented cutaneous nodular lesion, usually found on the sun-exposed areas of the skin, with far fewer such lesions occurring in covered areas. The most common sites are the trunk in men and the legs in women. Major risk factors for nodular melanoma include the presence of multiple dysplastic nevi, positive family history, light colored skin with an inability to tan, and excessive sun exposure. Nodular melanoma is known to present with greater thickness than the other subtypes of melanoma, therefore, it often carries a poorer prognosis [5]. Even in its early stages, it has the potential to metastasize to the vital organs [6]. Herein, we present a patient with melanoma which was associated with highly invasive and aggressive behavior.

## Case presentation

**Figure 1:**
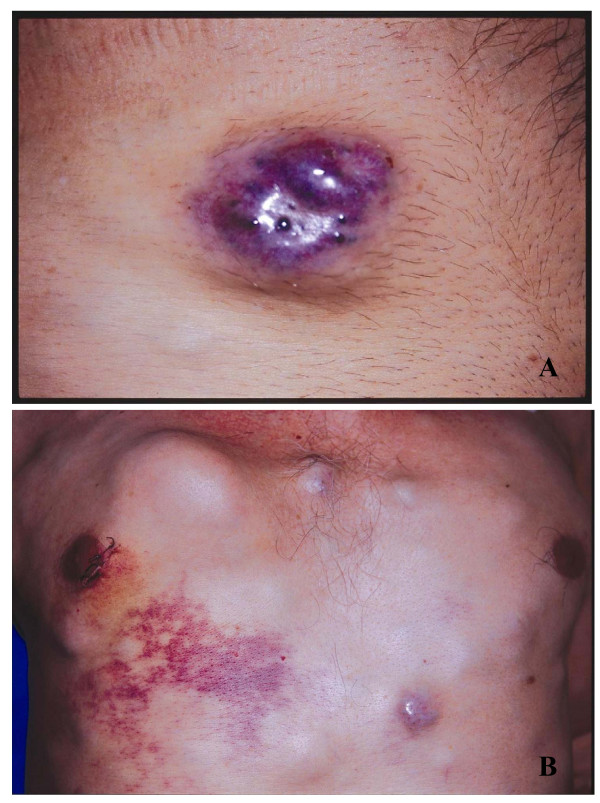
**(A) Clinical image shows a blue red nodule on the skin over the trunk**. (B) Clinical image shows multiple, dark colored, firm, nodular skin lesions varying in size.

**Figure 2:**
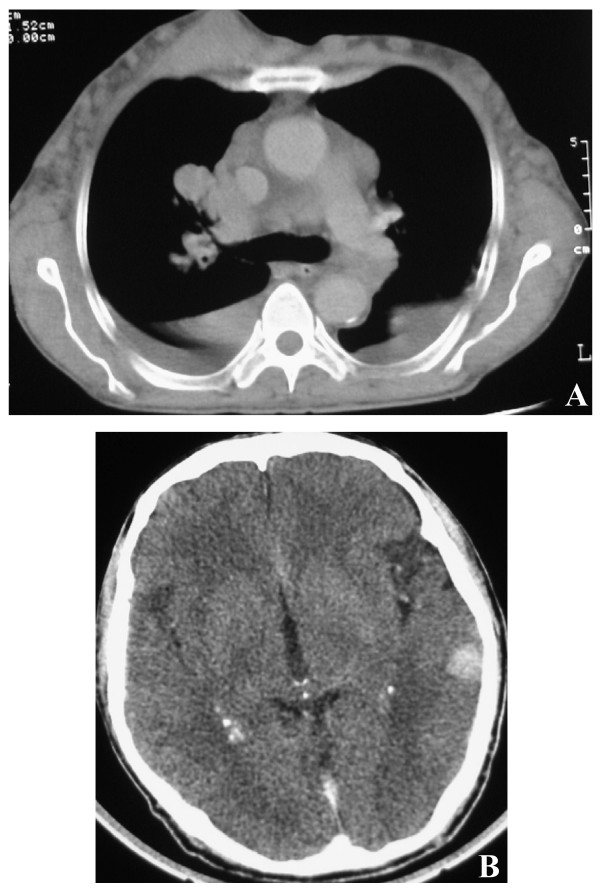
**(A) Computed tomography image shows a lobulated, irregularly shaped mass 7 × 4.5 cm in diameter with central hypodensity in the right atrium**. (B) Computed tomography image shows a brain metastasis.

**Figure 3:**
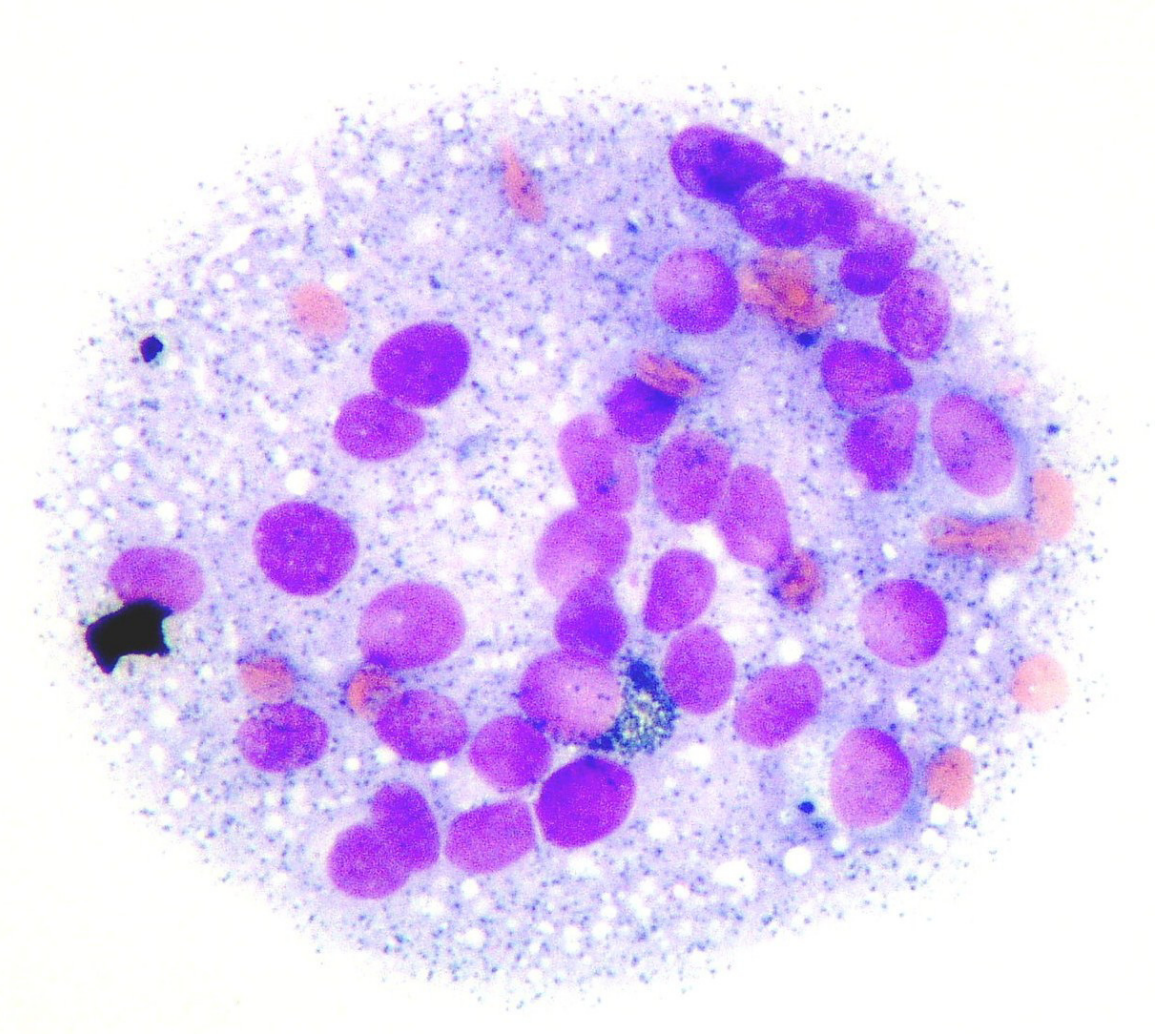
**A cluster of melanin pigment containing melanoma cells is observed in the fine needle aspiration cytology (hematoxylin and eosin staining, 100×)**.

A 51-year-old man was admitted to our hospital with multiple enlarging masses in the inguinal and thoracal areas of2 months' duration, which progressively enlarged and spread around the whole body. In the 2 weeks before presentation, the lesions had became darkly pigmented, suggesting melanoma. At the time of presentation, he complained of dyspnea, cough, fever and night sweats. He had a smoking history of 20 cigarettes a day for the last 30 years. Dermatological examination revealed multiple, dark colored, firm, nodular lesions varying in size (Figure 1A, B). Rhonchi over the whole lung area were noted on auscultation. The results of routine laboratory studies of blood and urine were normal except for raised ESR (98 mm/hour), LDH (1311 U/ml) and uric acid (12.8 mg/dl). Peripheral blood smear and bone marrow examinations were normal. Chest X-ray showed the presence of heterogeneous opacities in the right lung field suggestive of multiple pulmonary metastases. Contrast-enhanced computed tomography of the abdomen demonstrated a low density soft tissue lesion 7 × 4.5 cm in diameter with irregular margins, located within the right atrium, which was also consistent with the presence of metastases (Figure 2A). Contrast-enhanced computed tomography of the thorax showed conglomerated mediastinal lymph nodes and numerous nodular pulmonary lesions, findings that suggested the presence of metastases. Pleural and pericardial effusions were also present as well as numerous subcutaneous lesions in the chest wall and brain metastases confirmed by computed tomography of the brain (Figure 2B). On fine needle aspiration, the nodular lesions were diagnosed as melanoma (Figure 3). Although the classification was not based on histological examination, the appearance of the masses and the clinical findings of the patient were consistent with the diagnosis of nodular melanoma. Temozolomide was administered for 5 consecutive days at a daily dose of 150 mg/m^2^/day but the patient died of respiratory failure within a week after diagnosis.

## Discussion

Nodular melanoma is more common in men than women. The trunk is a common site and a discrete nodule with dark black/brown pigmentation is typical. Ulceration and bleeding are common complications. Nodular melanoma has a peak incidence around 50 years of age [5]. It arises in normal skin or in a precursor lesion, but without an intervening radial growth phase. A widely accepted histopathological definition of nodular melanoma is a melanoma that lacks an in-situ component beyond three rete ridges of the invasive vertical growth phase; thus, even in its early stages, nodular melanoma has the potential to metastasize [6,7]. Acral lentiginous melanoma is most frequent in the 60 to 70 year age group. It was so named because of its predilection for acral (distal) areas of the body, particularly the palms, soles and subungual areas, and its distinct radial or "lentiginous" growth phase. Its diagnosis is described as being based on its histological, intradermal features showing a diffuse proliferation of large atypical melanocytes along the epidermal-dermal junction which is dispersed in a lentiginous pattern with marked acanthosis and elongation of the rete ridges. Acral lentiginous melanoma is the only sub-type of melanoma that occurs at the same rate in all races, predominantly on an area that seldom receives much sun exposure. It has been suggested that the etiology is different from that of nodular melanoma or that sun exposure is a lesser risk factor than melanoma elsewhere. Also, various histopathologic features including nodular and acral lentiginous subtypes, vertical growth phase, high mitotic activity and the presence of microscopic satellites are associated with poor prognosis [8]. Metastatic melanoma usually involves draining lymph nodes and occasionally adjacent skin first, but eventually metastasizes to distant visceral sites. The skin and subcutaneous lymph nodes (59%) are most commonly involved followed by lung (36%), brain (20%), liver (20%), bone (17%) and others (12%) [6,7]. In our patient, metastatic lesions were seen in the lungs, pleura, heart and brain at the time of diagnosis. Although the diagnosis was not confirmed histologically, widespread metastases that developed in the patient within a short period of time strongly suggested melanoma. In the literature [6,7], melanoma is reported to develop metastases in every organ. Similarly, the patient developed rapidly progressive vital organ metastases.

The Breslow thickness is the most important prognostic variable. Tumors of greater. Breslow thickness are more likely to invade lymphatic or blood vessels allowing a route of passage for distant spread (Table 1). The number and localization of metastases are useful for assessing disease stage and response to therapy. Patients with cutaneous, nodal, or gastrointestinal metastases have a median survival time of 12.5 months; those with pulmonary metastases have a median survival time of 8.3 months; and in those with liver, brain, or bone metastases, the median survival is 4.4 months. The median survival time is 7 months in patients with a single metastatic site, 4 months with two organ sites and 2 months with three or more metastatic sites. Similarly, the 12-month survival rate is 36% with a single metastatic site, 13% with two organ sites, and 0% with three or more metastatic sites [7]. The patient's Breslow thickness was greater than 4 mm. In our patient, the number of metastases was more than three and he died 2 months after the onset of his illness, as expected.

**Table 1:** Prognosis according to Breslow thickness in melanoma

	Breslow thickness	5-year survival
	In situ	90–100%
Stage I	< 1 mm	80–90%
Stage II	1–2 mm	70–80%
Stage III	2.1–4 mm	60–70%
Stage IV	> 4 mm	50%

Despite research, no consensus has been reached as to the optimal management, There is level I evidence for the treatment of stage III and stage IV patients. Traditionally, management of melanoma metastatic to distant sites involves either a single-drug or multi-drug chemotherapeutic regimen. However, complete response rates have been poor (< 6%) with a minimal increase in the median survival [9,10]. Chemotherapy was administered immediately after the diagnosis of melanoma was suggested, but he died of respiratory failure because of the rapidly progressive course of disease with widespread pulmonary, brain, heart and cutaneous metastases.

## Conclusion

Tumor thickness, level of invasion, and number of involved nodes are the most powerful prognostic indicators in nodular melanoma. As described in our patient, nodular melanoma has a strong tendency for widespread dissemination and metastasis to vital organs. Despite increased therapeutic options for the treatment of advanced melanoma, the results are disappointing in patients with widespread metastases.

## Abbreviations

ESR: erythrocyte sedimentation rate; LDH: lactate dehydrogenase

## Consent

Written informed consent was obtained from the patient for publication of this case report and any accompanying images. A copy of the written consent is available for review by the Editor-in-Chief of this journal.

## Competing interests

The authors declare that they have no competing interests.

## Authors' contributions

This report reflects the opinion of the authors and does not represent the official position of any institution or sponsor. MAE was responsible for reviewing previous research, journal hand searching, and drafting the report. IK and EK were responsible for provision of published trial bibliographies, and preparing photographs. YB contributed to the final draft of the manuscript and analysis of relevant data. IA was responsible for project coordination. All authors read and approved the final manuscript.
